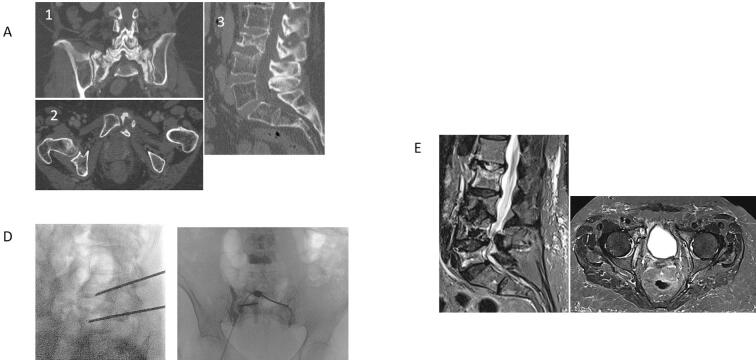# Pelvic insufficiency fractures after intensity modulated radiation therapy combined with chemotherapy for cervix carcinoma: Incidence and impact of bone mineral density

**DOI:** 10.1016/j.ctro.2023.100650

**Published:** 2023-06-12

**Authors:** Agathe Duranson, Vincent Thevenet, Frédéric Guyon, Guillaume Babin, Coriolan Lebreton, Tiphaine Renaud, Anne-Lise Gaillard, Quentin Dupuy, Wafa Bouleftour, Nicolas Magne, Adeline Petit

**Affiliations:** a Department of Radiation Oncology, Institut Bergonié, Comprehensive Cancer Center, Bordeaux, France; b Department of Statistics, Institut Bergonié, Comprehensive Cancer Center, Bordeaux, France; c Department of Surgery, Institut Bergonié, Comprehensive Cancer Center, Bordeaux, France; d Department of Medical Oncology, Institut Bergonié, Comprehensive Cancer Center, Bordeaux, France; e Department of Diagnostic Radiology, Institut Bergonié, Comprehensive Cancer Center, Bordeaux, France; f Department of Medical Physics, Institut Bergonié, Comprehensive Cancer Center, Bordeaux, France; g Department of Medical Oncology, North Hospital, University Hospital of Saint-Etienne, Saint-Etienne, France; h Cellular and Molecular Radiobiology Laboratory, Lyon-Sud Medical School, Unité Mixte de Recherche CNRS5822/IP2I, University of Lyon, Lyon, France

**Keywords:** Pelvic Insufficiency Fractures, Intensity Modulated Radiation Therapy, Locally advanced cervical cancer, Bone Mineral Density, Predictive factors

## Abstract

**Background and Purpose:**

The aim of this study was to evaluate the incidence and predictive factors of Pelvic Insufficiency Fractures (PIFs) occurring after Intensity Modulated Radiation Therapy (IMRT) combined with chemotherapy for locally advanced cervical cancer (CC).

**Material and methods:**

Medical records of patients receiving radio-chemotherapy with IMRT between 2010 and 2020 for advanced CC were reviewed. PIFs were detected during follow-up on pelvic Magnetic Resonance Imaging (MRI) or Computed Tomography (CT). The cumulative incidence rate of PIFs and its confidence interval were calculated at 2 and 5 years of follow-up. Pre-therapeutic Bone Mineral Density (BMD) (g/cm^3^) was evaluated on CT simulation for sacrum and the fourth lumbar (L4) vertebrae. Sacrum dosimetric parameters (V30Gy, V40Gy, D50%, Dmean) were analyzed.

**Results:**

136 patients were included. The median follow-up was 4.4 years. Median dose of D50% and V40Gy sacrum were 35.2 Gy (20.6–46.4) and 32.2% (7.2–73.4) respectively. The 2-year and 5-year cumulative incidence rates were 15.7% (95% CI: 9.88–22.71) and 22% (95% CI: 14.58–30.45) respectively. Median time interval between RT completion and PIFs’ detection was 11.5 months (IQR: 7.4–22.3). Univariate analysis showed that older age (p < 0.01), postmenopausal status at baseline (p < 0.01), and lower sacral and spinal BMD at baseline (respectively p < 0.001 and p < 0.01) were significantly associated to all sites of PIFs, and lower sacral BMD with sacral fractures (p < 0.001).

**Conclusion:**

Post-IMRT PIFs were detected in 18.4% of patients with locally advanced CC. Individual predisposing factors as older age, postmenopausal status, decreased bone density on the CT simulation were mainly predictive.

## Highlights

• 18.4% patients of this cohort experienced Pelvic Insufficiency Fractures (PIFs), which indicate that this complication is not so rare affecting one in 5 patients.
• Univariate analysis showed that older age (p < 0.01), postmenopausal status at baseline (p < 0.01), and lower sacral and spinal bone mineral density at baseline (respectively p < 0.001 and p < 0.01) were significantly associated to all sites of PIFs.
• The novelty of this study was the calculation of BMD through computed tomography planning for radiotherapy.

## Introduction

Radiotherapy (RT) combined with weekly radio-sensitizing platin-based chemotherapy followed by brachytherapy is considered as the gold standard treatment for locally advanced cervical cancer (CC) [1], [2]. Otherwise, Intensity modulated Radiation Therapy (IMRT) decrease gastrointestinal and urinary toxicities [3] and is the standard radiation technic for cervix carcinoma in western countries [1]. CC incidence and mortality rates have declined in the past few decades [4], [5]. Therefore, more attention was given to the late effects of the treatments [6], [7], [8], [9].

Pelvic insufficiency fractures (PIFs) represent a less common late toxicity after pelvic RT and their prevention was historically an optional endpoint in clinical studies. Incidence rates of RT-induced PIFs in CC are ranging from 0.45% to 89% [10], [11]. Heterogeneity of evaluation modalities (clinical *versus* radiological), inclusion of patients regardless of the primitive pelvic cancer or the administration of concomitant chemotherapy, and the use of different radiation techniques, could explain this heterogeneity. PIFs occur on underlying weakened bone with decreased elastic resistance after normal physiological stress, as opposed to traumatic and malignant fractures [12], [13]. Some studies suggest that radiation modify the bone matrix by damaging osteoblasts cells and reducing the vascular supply [14], [15]. Thus, RT is considered to be an important risk factor in PIFs development. Postmenopausal status, type II diabetes mellitus, lower Body Mass Index (BMI), older age and bone weakening diseases such as osteoporosis are other potential risk factors[16]. As known, osteoporosis significantly increases fracture risk [17]. However, scare studies have evaluated the association between bones mineral density (BMD) of pelvic bones before RT and postradiation PIFs [18], [19]. In fact, pretreatment bone density is frequently not available, regular methods to assess BMD increase radiation exposure and could delay curative treatment. Consequently, new modalities of BMD assessment are required. Furthermore, few studies have investigated the relation between Dose-Volume Histogram (DVH) parameters and the risk of PIFs in patients undergoing IMRT for advanced CC [20], [21]. Herein, the aim of the present study was to evaluate the incidence of PIFs after pelvic IMRT with concomitant chemotherapy and to analyze individual risk factors like quantitative assessment of BMD or dosimetric parameters.

## Methods and materials

### Study design

The study is a retrospective cohort of patients diagnosed with locally advanced CC treated with concomitant chemo-IMRT in a single institution from 01/01/2010 to 12/31/2020.

### Study population

All adult females with history of locally advanced CC treated by concomitant radio-chemotherapy were enrolled in this cohort through patient medical file. Concomitance chemotherapy was defined by a time interval of one month between the beginning of the first course of chemotherapy and the first session of radiation therapy. Only patients treated with IMRT either with Volumetric Arctherapy (VMAT) or Helical Tomotherapy® were included for the analysis.

All patients had a baseline imaging during the four months prior to the first session of RT. Patients with preexisting unhealed fractures of the pelvic bones at baseline were excluded. Patients with metastases to distant organs at diagnosis (defined by stage IVB in International Federation of Gynecology and Obstetrics (FIGO) 2009) [22], those who had already received pelvic irradiation, those who received neoadjuvant chemotherapy and those treated by three-dimensional conformal RT were also excluded. Furthermore, patients with pelvic metal implants or patients without follow-up imaging were not included.

### Treatment characteristics

Whole pelvis irradiation was delivered in IMRT. Median pelvic prescribed dose was of 45.0 Gray (Gy), 1.8 Gy per fraction, over five weeks. Patients with positive *para*-aortic nodes received RT with extended fields to lomboaortic area. Boost treatment for involved nodes could be applied as simultaneous integrated boost or as sequential boost, respectively to 55 and 65 Gy, except when nodes were previously removed during lymphadenectomy. All patients received concomitant weekly intravenous platinum-based chemotherapy. Brachytherapy was the technical reference for the tumor boost. Radical hysterectomy was performed when patients could not undergo brachytherapy or in case of residual disease after brachytherapy.

### RT planning and dose volume histogram (DVH) constraints

The contrast enhanced CT (CECT) simulation scan images were taken with 2.5 mm (mm) slice thickness reconstruction. A uniform standard for target and organ at risk delineation was used. Tumoral clinical target volume (CTV-T) contained the primary tumor, whole cervix, uterus, parametrial tissue, half superior of vagina and nodal target (CTV-N) contained presacral, common, external, and internal iliac and obturator region. Common iliac and *para*-aortic node region were included in cases of metastasis nodes spread to these regions. The planning target volume (PTV) included CTV-T and CTV-N with respectively 8 and 5 mm added margins in all directions. For bones delineation, five segments were considered, added to bone marrow volumes: sacrum, right and left ilium, and right and left pubis. Sacral foramina were included within the sacral contour when surrounded by bone on the CT slice. Coccyx was excluded from the sacral contour.

Dosimetric constraints to organ at risk were bladder V30Gy < 40%, rectum V40Gy < 40%, intestine V40Gy < 200 cc (cc). Optimal dose limitation strategy for bone marrow sparing was established according to Mell *et al*. publication [23].

### BMD assessment

**Fig. 1:**
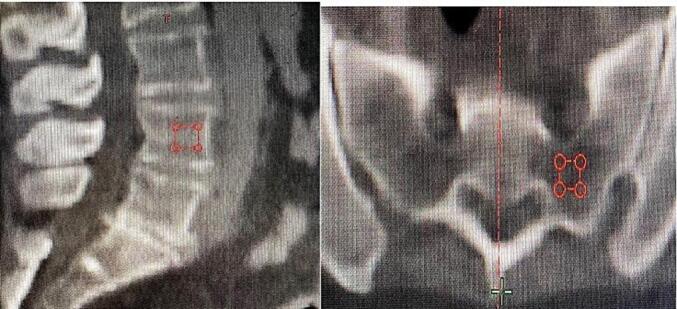
Representative case showing the Region of interest (ROI) placement on L4 and on the left portion of the sacrum.

Pretreatment BMD was measured through the CT simulation. A square Region Of Interest (ROI) of 10 mm of height was placed on the fourth lumbar vertebrae in the sagittal plane, in the ventral half of the trabecular compartment of the vertebrae as described by Kurrumeli *et al*. [19]. Two other ROIs of 5 mm of height were placed in the right and left side of the sacrum using visually the lowest density on bone windows. A specific tool on Eclipse Software^TM^ was used to show mean radiodensity values of the bone in Hounsfield Units (HU), corresponding to the volume in the square ROI. A representative case is shown on Fig. 1. The mean of the left and right sacral values was used for analysis, as well as the lumbar value. The HUs were then converted into mass density through the CT calibration curve obtained by the department physicist during the treatment planification system commissioning by imaging a phantom with different inserts with known mass density.

### Data collection

Data were collected and managed using the Research Electronic Data Capture (REDCap) tool. Demographic, clinical and pathologic characteristics were collected from the patient’s electronic medical record.

### Pifs diagnosis and follow-up

Follow-up was carried out with pelvic Magnetic Resonance Imaging (MRI), routinely at 6 and 12 months after the end of treatment then annually. Pelvic Computed-Tomography (CT) scans or Positron Emission Tomography CT (PET-CT) were performed in cases of contraindications to MRI. PIFs was defined as hypointense lines on T2-weighted or T1-weighted MRI sequences, surrounded by bone marrow oedema, hypointense on T1-weighted sequences, hyperintense on T2-weighted sequences. Diagnosis was determined on CT as low-density lines, with or without surrounding bone sclerosis. Traumatic or metastatic lesions were excluded by history and radiological appearances.

Fracture sites were categorized into five subgroups: spinal, sacrum, left and right ilium, and pubic bone. Fractures involving the sacroiliac joint were categorized as sacral fractures. In addition, locations and numbers of fractures were collected.

### Statistical analysis

Qualitative variables were described using numbers and percentages. Quantitative variables were described using medians, range and interquartile. The cumulative incidence rate of PIFs and its confidence interval were calculated at 2 and 5 years of follow-up. Association between PIFs, individual factors and radiation characteristics were analyzed by univariate Cox regression analysis. P-values<0.05 were considered statistically significant. All statistical data analyses were performed using SAS version 9.4.

### Standard approvals and patient consent

All patients received written information and Institutional Review Board approval was obtained.

## Results

### Patient demographic and clinical characteristics

**Fig. 2:**
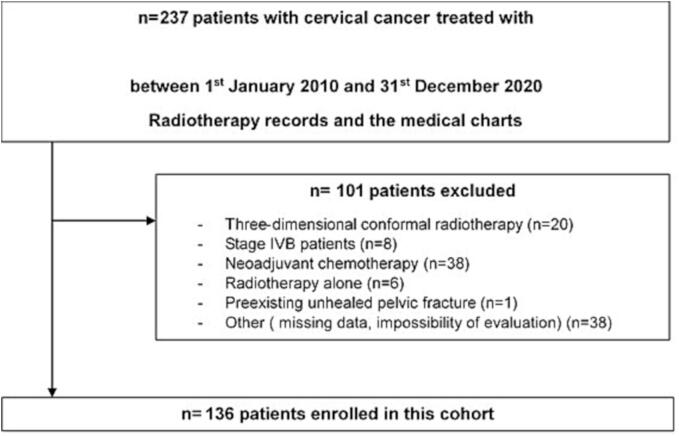
Flow chart presenting the selection process of the cohort study.

A total of 136 patients diagnosed with CC and receiving a concomitant chemo-RT by IMRT were enrolled (Fig. 2). The median age was 49 years (IQR: 42–59.5, range: 28–82 years). Regarding to menopausal status, 59 patients (43.4%) were postmenopausal at diagnosis and only one of them received Hormone Replacement Therapy (HRT) prior to RT treatment. Osteoporosis status was available for 73 patients (53.7%), and only one patient presented osteoporosis at diagnosis (0.7%).

More than half of patients (61.8%) had IIB FIGO 2009 stage tumor. Eighty patients (58.8%) had an involvement of regional lymph nodes at the time of diagnosis. Pelvic lymph node boost was performed on 54 patients (39.7%), with a median dose of 57.5 Gy (range 50.4–65.0) and *para*-aortic lymph node boost on 31 patients (22.8%) with a median dose of 57.5 Gy (range 50.0–67.0). An external radiation boost on the tumor was performed for 23 patients (16.9%) with a median dose of 21.6 Gy (range 10.0–25.2) whereas 108 patients (79.4%) underwent brachytherapy boost. Hysterectomy was performed prior to radio-chemotherapy on 7 patients (5.1%), and after the radiation on 27 patients (20.9% of the 129 patients remaining). Median sacrum V30Gy, V40Gy, and D50% were 71.9% (range 23.6–99.5), 32.2% (7.2–73.4), and 35.2 Gy (20.6–46.4), respectively. All clinical and demographic characteristics are summarized in Table 1.

**Table 1:** Patient, tumor, and therapeutic characteristics.

Study population (n = 136)	
**Patient characteristics**	n (%)
**Median age, year (range)**	49 (28–82)
**Median BMI, kg/m2 (range)**	24.0 (15.0–40.0)
**Preexisting osteoporosis** Yes No No data available	1 (0.7) 72 (52.9) 63 (46.3)
**Menopause at baseline**	59 (43.4)
**HRT at baseline**	1 (1.7)
**Active smoking at baseline** Yes No No data available	47 (34.6) 84 (61.8) 5 (3.7)
**Type 2 Diabetes mellitus**	4 (2.9)
**Tumor characteristics**	N (%)
**Histopathology** Adenocarcinoma Squamous cell carcinoma Other	20 (14.7) 111 (81.6) 5 (3.7)
**FIGO stage (2009)** IB1 IB2 IIA1 IIA2 IIB IIIA IIIB IVA	9 (6.6) 16 (11.8) 5 (3.6) 3 (2.2) 84 (61.8) 7 (5.1) 9 (6.6) 3 (2.2)
**Therapeutic characteristics**	
**Radiation technique** Helical Tomotherapy® Volumetric Modulated Arctherapy	88 (64.7) 48 (35.3)
**Median dose, Gy (range)**	45.0 (30.6–45.0)
**Nodal involvement**	80 (58.8)
**Lymph node boost** Pelvic lymph node boost Para-aortic lymph node boost	54 (39.7) 31 (22.8)
**Para-aortic extended field**	42 (30.9)
**Additional tumor boost**	23 (16.9)
**Sacrum DVH parameters** Median Sacrum Dmean, Gy (range) n = 107 Median Sacrum D50%, Gy (range) n = 107 Median Sacrum V30Gy, % (range) n = 107 Median Sacrum V40Gy, % (range) n = 107	34.8 (20.0–45.1) 35.2 (20.6–46.4) 71.9 (23.6–99.5) 32.2 (7.2–73.4)
**Brachytherapy**	108 (79.4)
**Hysterectomy** Prior to RCT Hysterectomy after RCT*	34 (25) 7 (5.1) 27 (20.9)
**Lymph node dissection**	107 (78.7)
**Median cycles of chemotherapy (range)**	5 (2–8)

N = sample size; BMI: Body Mass Index; HRT: Hormone Replacement treatment; RCT: radiochemotherapy;

*patients with hysterectomy performed prior to radiochemotherapy were excluded for the analysis.

### PIFs incidence rate

The median follow-up was 4.4 years among our population (95% confidence interval [CI]: 3.6–4.9). The 2-year and 5-year cumulative incidence rate of PIFs after RT were 15.7% (95% CI: 9.88–22.71) and 22% (95% CI: 14.58–30.45), respectively. The median interval time between the end of RT and PIF diagnosis was 11.5 months (IQR: 7.4–22.3 months, range 3.5–49.7 months). The median age of patients experiencing PIFs was 58 years (IQR 44–65, range 35–80).

Out of the 136 patients, 25 (18.4%) were diagnosed with PIFs located in the irradiated fields, with a total of 39 fractures. In 71.8%, 12.8%, 10.3% and 5.1% of cases, PIFs were located in sacrum, lumbar spinal vertebra, pubis and ilium, respectively. Interestingly, 22 of the 25 patients (92%) who experienced PIFs had at least one sacral fracture. Fractures were mostly asymptomatic (51.3% of total fractures), diagnosed either with MRI in 71.8% of cases or with CT in 28.2% of total fractures. Pain medication and cementoplasty were used for 28.2% and 17.9% of fractures, respectively. Of note, a cementoplasty was performed two times on the same fracture.

Bisphosphonates were introduced for 2.2% of all patients during the follow-up, one of whom had been diagnosed with PIF prior to the introduction. HRT was introduced for 7.4% of patients after the radio-chemotherapy, two of them were diagnosed with fracture, one prior and another one after the introduction. Ten patients had multiple fractures and one patient had 5 fractures (Annex. 1). This particular patient had two fractures on the pubic bone, one lumbar spinal vertebra fracture, one on left ilium and one on sacral bone.

### PIFs risk factors analysis

Univariate analysis revealed both age and menopausal status at baseline were significantly related to PIFs development with respectively Hazard Ratio (HR) of 1.28 ([95% CI: 1.10 – 1.50] - p < 0.01) and 3.31 ([95% CI: 1.43 – 7.69] - p < 0.01) (Table 2).

**Table 2:** Univariate analysis of risk factors associated with all-sites PIFs.

Characteristics	Unit/Modality		Hazard Ratio (HR)	95% Confidence Interval (CI)	p-value
AgeN = 136	5 years		1.28	1.10 – 1.50	**<0.01**
BMI at baseline*N = 136	kg/m^2^		1.52	0.33 – 7.13	0.59
	<1.5 years	<18.5 vs Normal			

	≥ 25.0 vs Normal	1.52	0.57 – 4.03	0.40
	>1.5 years	<18.5 vs Normal	0.83	0.16 – 4.29	0.83
≥ 25.0 vs Normal		0.45	0.08 – 2.49	0.45
Menopausal at baselineN = 136	Yes vs No		3.31	1.43 – 7.69	**<0.01**
Active smoking at baseline*N = 136	Yes vs No		0.93	0.34 – 2.51	0.88
	<1.1 year				
	>1.1 year		1.08	0.26 – 4.54	0.91
Spinal BMDN = 131	0.01 g/cm^3^		0.82	0.71–0.95	**<0.01**
Sacral BMDN = 131	0.01 g/cm^3^		0.65	0.54 – 0.79	**<0.001**
Radiation boost on tumorN = 136	Yes vs No		1.48	0.55 – 3.96	0.43
Radiation field extended to *para*-aortic area*N = 136	Yes vs No		0.83	0.30 – 2.32	0.72
	<1.8 year				
	>1.8 year		0.32	0.04 – 2.63	0.29

N = sample size; BMD: Bone Mineral Density; BMI: Body Mass Index;

* time stratification was performed after analysis of schoenfled residual.

Underweight and overweight BMIs at baseline were associated with an increased likelihood of PIFs during the first 1.5 years of follow-up but were not statistically significant (HR 1.52 [95% CI: 0.33 – 7.13] - p = 0.59 and HR 1.52 [95% CI: 0.57 – 4.03] - p = 0.40 respectively). Likewise, the radiation boost on tumor (HR 1.48 [95% CI: 0.55 – 3.96] – p = 0.43) was not statistically significantly linked to PIFs (Table 2).

**Fig. 3:**
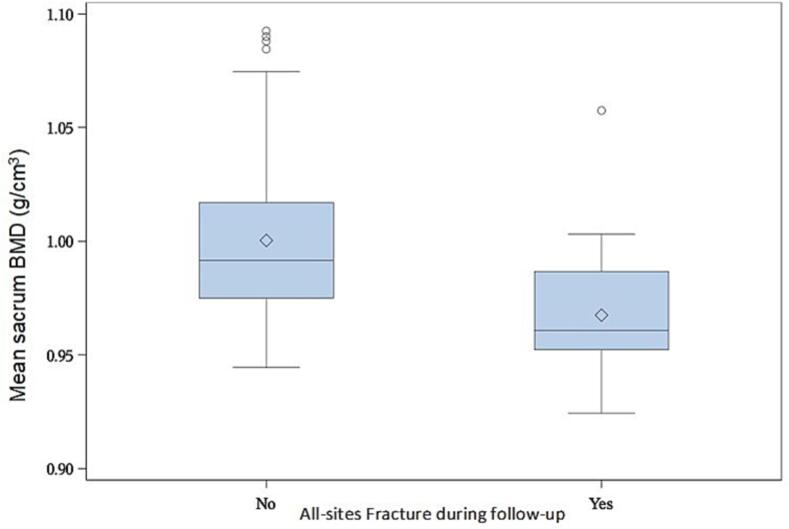
Boxplot shows the bone mineral density (BMD) of the sacrum in patients with a pelvic insufficiency fracture and in those without.

**Fig. 4:**
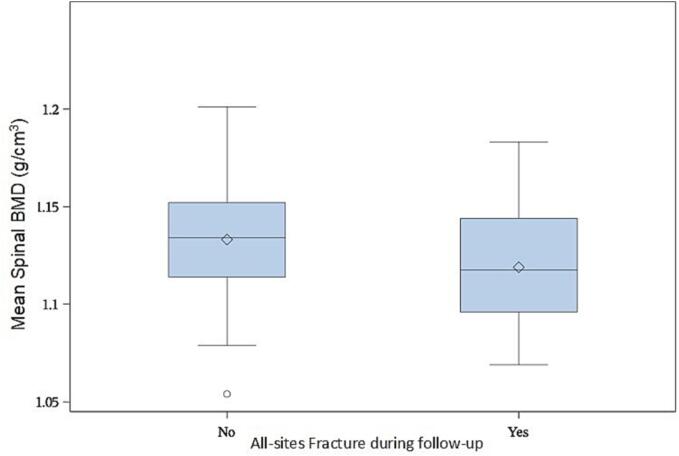
Boxplot shows the bone mineral density (BMD) of the lumbar vertebrae in patients with a pelvic insufficiency fracture and in those without.

**Fig. 5:**
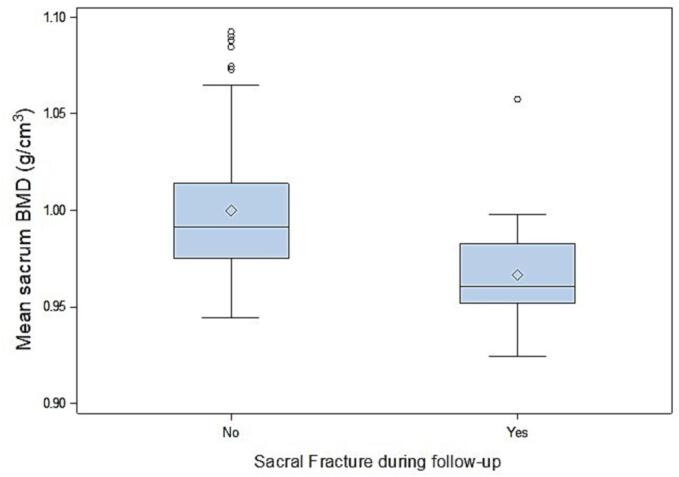
Boxplot shows the bone mineral density (BMD) of the sacrum in patients with a sacral insufficiency fracture and in those without.

Spinal and sacral BMD were both significantly associated with all-sites PIFs, an increase of 0.01 g/cm^3^ of density reduced the risk of all-sites fractures of respectively 18% for spinal and 35% for sacral BMD (HR 0.82 [95% CI: 0.71 – 0.95] – p < 0.01) and HR 0.65 [95% CI: 0.54 – 0.79] – p < 0.001, respectively) (Fig. 3 and Fig. 4). Higher sacrum BMD was also significantly associated with a reduction of sacrum fractures (HR 0.65 [95% CI: 0.53 – 0.79] – p < 0.001) (Fig. 5).

Dosimetric analyses were performed on 107 patients. Sacrum DVH values (V30Gy, V40Gy, Dmean, D50%) were not associated with all-sites fractures events (p = 0.07, p = 0.15, p = 0.28 and p = 0.10 respectively). Sacral dose values V30Gy and V40Gy were not significantly associated with sacral insufficiency fractures (p = 0.07 and p = 0.15, respectively).

## Discussion

This study allowed a PIFs incidence estimation in patients with locally advanced CC treated by IMRT and chemotherapy combination. Indeed, 18.4% patients of this cohort experienced PIFs, which indicate that this complication is not so rare affecting one in 5 patients. In this cohort, PIFs 5-year cumulative incidence rate was 22%, whereas Razavian *et al*. *meta*-analysis showed crude incidence rate of 9.4% [16]. This difference of estimation might be related to an inclusion of studies reporting only symptomatic PIFs, and thus inducing an underestimation of PIFs incidence. Although the criteria for diagnosing the pelvic fractures could be considered somehow more sensitive than other experiences, the incidence of 18.4% is in the line with the upper border of the first *meta*-analysis published on this topic, which indicated a point estimate of 14%, with 95 %CI 10%-18% [24]. Similarly, the location of the fractures is also in accordance with this *meta*-analysis (70% in the current study vs. 73.6% in the referred *meta*-analysis [24].

Likewise, a recent *meta*-analysis which pooled studies used MRI as a diagnosis tool reported a similar PIFs incidence rate as our study [25].

PIFs should also be considered as an early late complication. Indeed, the median time interval between the end of RT and PIFs occurrence was 11.5 months with 75% of fractures occurring in the first 2 years. These data are supported by by Bazire *et al*. [21] and Ramlov *et al*. [20] which reported a similar results. Regarding to PIFs location, more than 70% of PIFs were located within the sacrum, and 92% of PIFs patients had at least one sacral fracture [24], this could be due weight-bearing property of pelvic bone.

Postmenopausal status at baseline and age are the principal predisposing factors related to post-radiation PIFs occurrence reported in either retrospective [26], prospective studies [27] and in series with IMRT-only treatment [20], [21]. These two features are highly interrelated and are both known to be risk factors for osteoporosis [17]. Furthermore, our data showed that lower spinal and sacral BMD were significantly predisposing factors for developing post-radiation all-sites PIFs. In addition, higher sacral BMD was significantly associated with a reduction of sacral located fractures. A previous study which enrolled 59 patients treated with three-dimensional conformal radiotherapy, reported the same correlation through multivariate analysis but using indirectly CT simulation Hounsfield units HU (p = 0.020), with ROIs placed in lumbar vertebra, left and right sacrum [18]. Kurrumeli *et al.* presented for the first time a method to assess BMD via planning CTs in the case of radiation induced PIFs in CC, and showed comparable results for spinal and sacral BMD (p = 0.03), however fractures occurred in only in 9.6% of patients[19]. Previous studies have suggested a benefit of IMRT compared to three-dimensional conformal radiotherapy to spare bone and consequently decrease bone toxicity [28], [29], [30]. However, clinical results failed to demonstrate this benefit [16].

There is a growing interest in reducing the dose to the pelvic bones with the primary intention to save the bone marrow. Herein, we performed a bone marrow-sparing as described by Mell *et al.* [23] which could therefore indirectly induced a sacrum dose ballistic optimization. With a median D50% sacrum of 35.2 Gy (range: 20.6–46.4), a median Dmean of 34.8 Gy (20.0–45.1), and a median V40% of 32.2% (7.2–73.4), no statistically significant relation between DVH sacrum parameters and all-sites PIFs (p = 0.10, p = 0.07, p = 0.28 respectively) was observed. Ramlov *et al*. [20] found that D50% sacrum greater than 37.8 Gy was associated with an increased risk of PIFs in the group of patients over than 50 years old with an Odds Ratio (OR) of 4.73 (95% IC:1.33–16.8) (p = 0.016). Their model also suggests that a 5 Gy decrease on sacrum D50% from 40 Gy to 35 Gy would allow a reduction of the risk of PIFs occurrence around 22% to 45% [20].

Mir *et al*. [31] established a predictive model of PIFs demonstrating that a reduction of sacral V40Gy from 43.1% to 31.9% will reduce the risk of sacral insufficiency fractures from 8% to 14% depending of the age category. In addition, their nomogram suggests that a sacral V40Gy inferior to 30% could lead to a PIFs incidence lower than 20%. With a sacral median V40Gy of 32.2% and a crude incidence rate of 18.4%, our data are in concordance with Mir *et al.* findings. According to Bazire *et al*. [21] data, insufficiency fracture sites received a higher maximum dose of radiation compared to nonfracture sites (p = 0.045). Recently, Chopade *et al*. [32] evaluated feasibility of bone sparing on L4 and L5 vertebrae in patients undergoing post-operative pelvic IMRT by applying dose constraints to the bones, with a prescribed dose of 50 Gy in 25 fractions. Dose-response relationship was observed between radiation dose and bone mineral density loss [32]. Uezono *et al*. evaluated 2-years cumulative incidence at 32% with a median prescribed dose of 50.4 Gy at 1.8 Gy per fraction [18]. These data are consistent with actual recommendations to limit external radiation prescription to 45 Gy, considering reduction of late bowel morbidity and taking into account specific survival.

Future interests could concern proton therapy which is currently the subject of a Phase-II-Trial [33]. This study evaluate the differences in toxicities between photon therapy and proton therapy, both combined with chemotherapy for locally advanced CC, and could provide new data on PIFs incidence. The growing interest for this technique is due to hematopoietic progenitors’ sparing quality and therefore a potential immunosuppression reduction and an improvement of chemoradiation tolerance, safety and efficacy.

Finally, medicine prevention and PIFs therapeutic strategies remain to be determined. Indeed, the use of HRT as PIFs protective factor is associated with variable results. A protective relationship between HRT and PIFs for premenopausal women receiving HRT after the RT was observed [20]. In contrast, another study observed that patients receiving HRT prior to RT had a significantly higher 5-year incidence of PIFs, presuming that the protective effect of HRT ends at hormone treatment cessation.

The role of bisphosphonates in PIFs treatment and prevention remains unclear. Pamidronate tended to improve radiological healing of PIFs in vaginal and endometrial cancers (p = 0.11) [34]. The position of the American Society of Clinical Oncology (ASCO) regarding to the use of bisphosphonates for post-radiation fractures are yet to be determined as it could modify vascular environment already compromised on an irradiated bone [35]. Thereby, further studies are urgently needed to determine adequate pharmacological interventions [36].

In conclusion, the incidence of PIFs was not so rare affecting 18.4% of patients with CC. The novelty of this study was the calculation of BMD through planning CT for RT, making the present study, to our knowledge, one of the largest series published in PIFs and BMD assessment via planning CT. This BMD assessment technique could enable clinicians to evaluate PIFs’ risk prior to RT and therefore adapt the follow-up modalities to each patient by taking into account bone toxicity.

## Declaration of Competing Interest

The authors declare that they have no known competing financial interests or personal relationships that could have appeared to influence the work reported in this paper.

## Figures and Tables

**Supplementary figure 1:**